# Livestock Identification Using Deep Learning for Traceability

**DOI:** 10.3390/s22218256

**Published:** 2022-10-28

**Authors:** Hai Ho Dac, Claudia Gonzalez Viejo, Nir Lipovetzky, Eden Tongson, Frank R. Dunshea, Sigfredo Fuentes

**Affiliations:** 1 Digital Agriculture, Food and Wine Sciences Group, School of Agriculture and Food, Faculty of Veterinary and Agricultural Sciences, University of Melbourne, Melbourne, VIC 3010, Australia; 2 School of Computing and Information Systems, Faculty of Engineering and Information Technology, The University of Melbourne, Parkville, VIC 3010, Australia; 3 Faculty of Biological Sciences, The University of Leeds, Leeds LS2 9JT, UK

**Keywords:** cow identification system, deep learning in agriculture, computer vision, edge computing

## Abstract

Farm livestock identification and welfare assessment using non-invasive digital technology have gained interest in agriculture in the last decade, especially for accurate traceability. This study aimed to develop a face recognition system for dairy farm cows using advanced deep-learning models and computer vision techniques. This approach is non-invasive and potentially applicable to other farm animals of importance for identification and welfare assessment. The video analysis pipeline follows standard human face recognition systems made of four significant steps: (i) face detection, (ii) face cropping, (iii) face encoding, and (iv) face lookup. Three deep learning (DL) models were used within the analysis pipeline: (i) face detector, (ii) landmark predictor, and (iii) face encoder. All DL models were finetuned through transfer learning on a dairy cow dataset collected from a robotic dairy farm located in the Dookie campus at The University of Melbourne, Australia. Results showed that the accuracy across videos from 89 different dairy cows achieved an overall accuracy of 84%. The computer program developed may be deployed on edge devices, and it was tested on NVIDIA Jetson Nano board with a camera stream. Furthermore, it could be integrated into welfare assessment previously developed by our research group.

## 1. Introduction

For traceability purposes, farmers and producers are highly interested in animal recognition and identification. Conventional techniques to identify livestock involve microchips, ear tags, tattoos, and Radio Frequency Identification (RFID) collars, among others. However, most of these identification methods are prone to be lost and destroyed due to animal movement, direct contact, and bites. Furthermore, electronic devices can be hacked or physically exchanged between animals for fraudulent practices [1], and they can involve relatively high costs for large herds of animals [2,3,4,5]. Since these devices need the intervention of humans for data recording and maintenance, they tend to be time-consuming and unreliable due to tattoos fading and tags being lost [6]. In pigs, researchers have reported a loss rate of ear tags within 5–60% [7], 19% implants, and loss or damage of 23% of electronic identification devices at slaughter [6]. In cattle, a loss of 20% after the first five years has been reported, with an extra 20% loss per year in the following years [8]. Recently, researchers have used new digital approaches based on computer vision (CV), deep learning (DL), machine learning (ML), and artificial intelligence (AI) to automate and monitor livestock recognition and identification for different animals, such as bears [9], sheep [10], giant panda [11], and cattle [2,12]. Specifically, these approaches use face recognition and muzzle pattern recognition [4] that are unique for each individual, similar to fingerprints, patterns in the body of dairy cows [3], or iris recognition [13]. The latter studies have shown that CV, DL, ML, and AI technologies have great potential to be applied to livestock identification and management as non-invasive and efficient automated methods.

Therefore, this study aimed to develop a deep learning model to recognize and identify dairy cows from a robotic dairy farm in Australia using face recognition. The pipeline presented in this paper can be applied to other livestock. The outcome of this research will show the maturity and readiness of the advancing state-of-the-art computer vision models to benefit the livestock industry, especially for automated traceability. The proposal prototype is designed with flexible options for deployment and integrating new features in the future that potentially can replace many physical tags and wearable sensor devices.

## 2. Materials and Methods

### 2.1. Data Collection from Dairy Cows

**Figure 1:**
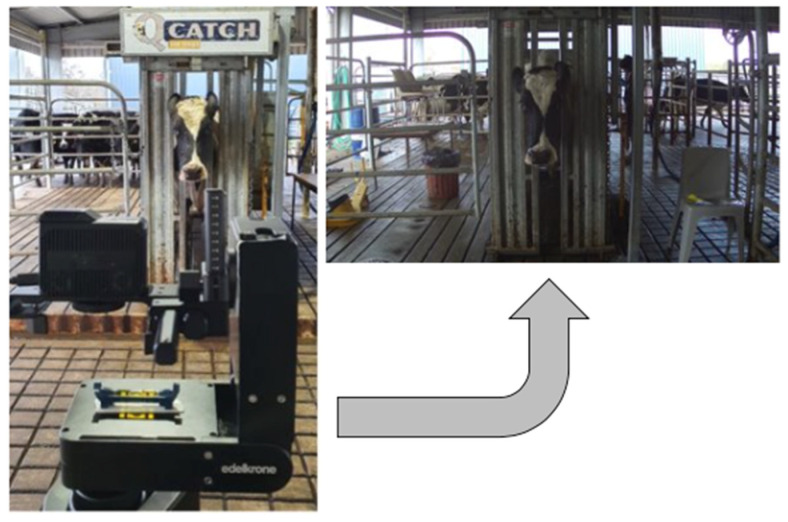
A sample setup of a recording session of the cattle videos.

One-minute videos from N = 102 different Holstein-Friesian cows located at the Robotic Dairy Farm belonging to the Dookie College, The University of Melbourne (UoM), Victoria, Australia (36°38′ S, 145°71′ E), were recorded over four days during the winter of 2021 (total 281 video clips; ~505,800 video frames). For 89 cows, at least two videos were recorded, while the other 13 had only one video. Each video consisted of a single identity as the main target appearing in the middle of the frame throughout the entire video (frames per second: 30 fps, average duration = 60 ± 19 s). Cows were held in a crush, so their heads moved freely (Figure 1). Videos were recorded using a FLIR DUO PRO (Teledyne FLIR LLC, Wilsonville, OR, USA). This camera can record 4K resolution RGB and thermal infrared videos. However, for this study, only the RGB videos were used. The camera was located ~2.5 m away from the crush to capture the face of the cows (Figure 1). All animal handling and welfare protocols followed in this study were approved by the Animal Ethics Committee of The University of Melbourne (Ethics ID: 2021-21466-18833-5).

### 2.2. Application Pipeline

**Figure 2:**
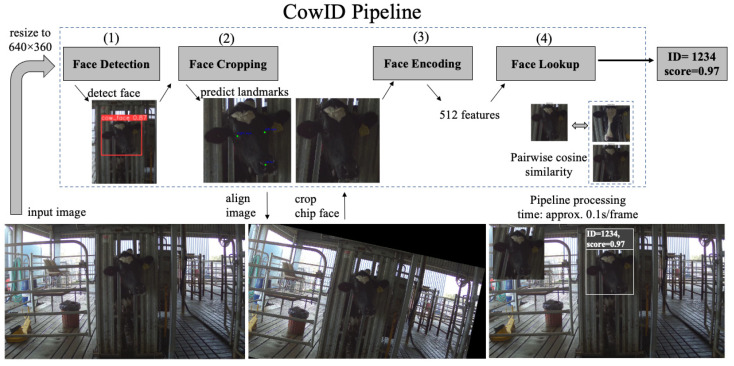
Diagram showing the pipeline of the cows recognition and identification system.

The pipeline (Figure 2) comprises four fundamental steps: (i) face detection, (ii) face cropping, (iii) face encoding, and (iv) face lookup. These components have been used in other deep-learning pipelines for face recognition [9]. Although naming these basic steps can differ from one paper’s terminology to another, they all share similar functionality to solve the face recognition problem [14]. The pipeline loads a still image and outputs a matched identification (ID) number of a detected cow with an associate confidence score. There are three deep learning models involved in the first three steps, respectively: (i) Face detector, (ii) Landmark predictor, and (iii) Face encoder.

In the face detection step, the Face detector model localizes the image coordinates of the cow’s face, adjusts the coordinates into a square shape, crops the face, and sends it to the Landmark predictor in the face cropping step. Landmark predictor, in the face cropping step, identifies markers on the squarely cropped face, such as the eyes and tip of the nose, to subsequently use these markers as anchors to help align the face into a portrait position, called a face image chip [14] or chip face [9] having both eyes on a line parallel to the x-axis. The portrait face is then cropped into a square shape and passed to the face encoding step to extract embedding features. The Face encoder model encodes the face into a vector representation called face embedding features in the face encoding step. These face embedding features are distinctive and unique to an individual, upon which the machine relies to distinguish one identity from the others. In other words, face embedding features of the same individual would be very similar to each other and different compared to embedding features of other individuals. In the last step, the face lookup algorithm ranks all the pairwise similarity scores between the embedding features of the input chip face with all the other embedding features of faces in the database. It outputs the face ID with the highest similarity score. The similarity score of a pairwise comparison spans the range [−1, 1], with 1 being a perfect match and −1 being the exact opposite. The returned ID and its associated similarity score from the face lookup step are the final output of the pipeline for the detected face in the input image.

A threshold, *T*, value acts as a level of confidence that can be introduced to the face lookup step to help filter out returned IDs with a low similarity score. If the similarity score is greater than *T*, it is an accepted returned ID; otherwise, the ID is considered “unidentified”. The “unidentified” ID may mean two things: the input chip face belongs to a new ID that does not exist in the database, or the similarity level is not high enough for the system to claim the returned ID is a correct match. In this case, the recognition system can be configured to return the closest *L* candidate IDs, according to the similarity score ranking, and end-users might examine either all *L* candidates or just the top R candidates (R ≤ *L*) or simply have to manually check the subject’s ID based on other physical tag or collar.

This pipeline can process video sequences by treating the video frames as individual still images. The video procedure uses a fixed-size buffer (i.e., 10, 20, or up to 50) to hold sampling every *n* number of frames (*n* used in this paper is six). It outputs the ID with the most occurrences as the representative ID across each frame in the buffer. The output score [0, 1] of the buffer represents the frame ratio in the buffer where the output ID was selected. Similar to the pipeline, there is also a threshold to obtain only highly confident outcomes. As the term “threshold” is used in multiple contexts, a prefix “pipe” or “buffer” is added to clarify the associated location of these terms in use, such as “pipID, pipScore, pipThreshold” for the pipeline in a single image processing and “bufID, bufScore, bufThreshold” for the buffer used in video processing.

All the annotations in this study were generated using the Computer Vision Annotation Tool (CVAT) [15]. Model training and testing were performed on NVIDIA GeForce GTX 2080 graphic card. The evaluation metrics of the pipeline follow the Face recognition vendor test for the identification task of the National Institute of Standards and Technology, US Department of Commerce [16].

### 2.3. Training and Testing Data

As 89 cows had at least two videos, one video of each cow was selected as a training video to extract data for the models’ training and validation, which populated the enrolled cow ID in the database. The other video, called the testing video, was saved to test the whole pipeline’s performance. The 13 cows with only one video were used to assess how the pipeline responds to a novel face, also considered testing videos. In short, there were 89 videos for training and 102 videos for testing. To simplify the terminology, a cow ID present in the database is referred to as “mate”, while cows unenrolled in the database are referred to as “non-mate” [16].

In this study, the training and testing datasets were novel with respect to any available public datasets, so the data generation played a critical part in the overall performance outcome. Although all three deep learning models used in this study were trained on popular public datasets such as ImageNet [17] or COCO [18] datasets (in which cattle were among the training classes), they did not explicitly map to dairy cow’s facial features. Since these models are related in a sequential flow along the pipeline, they need to be trained in order, starting with the Face detector, followed by the Landmark predictor and Face encoder. Data used to finetune the models was sampled from the training videos.

Face detector, the first model in the pipeline, was finetuned on a total of 1780 randomly sampled frames from training videos where the cow’s face appeared in the middle of the frame. That is equivalent to 20 samples per individual cow. To retain a clear view of both eyes and the tip of the nose for the Landmark predictor in the next step, only the cow’s head that showed a clear view of those critical points was annotated with a bounding box. In contrast, frames were ignored and not annotated, where the cow’s head was turned to a side. Annotated data was split into training and validation sets with a ratio of 80/20, respectively. The face’s bounding box predicted by the Face detector was post-processed to become a square shape, and the face was also cropped accordingly before passing to the Landmark predictor.

The Landmark predictor intakes generated data from the face detection step for finetuning. To avoid using the same samples for training the Face detector, each training video was randomly sampled for another 20 frames. That comprised 1780 samples in total. These samples were put through the face detection step to crop the cow’s head into a square shape, ready for annotation and retraining of the Landmark predictor. Each face image shows three crucial points: the left eye, the right eye, and the tip of the muzzle. Training and testing datasets were split with a ratio of 80/20. As the Landmark predictor is a part of the face cropping step, the predicted facial landmarks were used as anchors to rotate the image to have the face in an aligned portrait position. An aligned portrait position is defined as the angle being zero degrees between a line connecting both eyes and the x-axis; in other words, both eyes are paralleled with the x-axis or the ground. When the face is aligned, it is cropped in a square shape. The aligned cropped square shape of the face is called ‘chip face’ and is passed to the next step.

The finetune Face encoder model dataset is a set of chip faces generated by the formerly trained face detector and landmark predictor. In some videos where the chip faces were extracted, there are a few consecutive frames where the cow head’s movement is not noticeable. If chip faces were sampled among these frames, it would result in too many chip faces in the database that look almost identical. Hence, capturing frontal faces from various angles is preferred to achieve sample diversity for a given identity. A new chip face sample of identity is considered eligible to be saved into the database when its pairwise cosine similarity with all previously saved identity features is below a threshold of 0.9. The MobileNetV2 model [19], pretrained on the ImageNet dataset [17], was used as a feature extractor. To achieve a decent amount of training and validation data, each video sampled 60 faces using the above method. From these, 40/60 were reserved for finetuning, and 20/60 were reserved for validation, which comprised 5340 chip faces in total, 3560 faces for training, and 1780 faces for validation. After finetuning the Face encoder model, it is necessary to build a database to validate the face lookup step and the whole pipeline. A subset of the first 25/40 training faces per identity was selected to enroll on a database comprising 2225 chip faces representing 89 cow mates. Then, the Face encoder was evaluated along with the face lookup step, equivalent to an overall validation of the whole pipeline on the training video set.

To assess the performance of the pipeline, the testing videos are separated into two testing sets: one closed set and one open set. The closed-set had a population of 89 cow mates, each with 100 faces sampled once every 200 ms (one sample every six frames) across 20 s per testing video, which adds up to 8900 chip faces. The testing set was larger than the training/validation set because it is not restricted by the sampling method used to select the chip face for the Face encoder model above. The open-set had a population of 13 cow non-mates novel to the pipeline. Faces were sampled the same way as the closed-set with 1300 chip faces, 100 faces sampled once every 200 ms, across 20 s per testing video.

### 2.4. Face Detector

**Figure 3:**
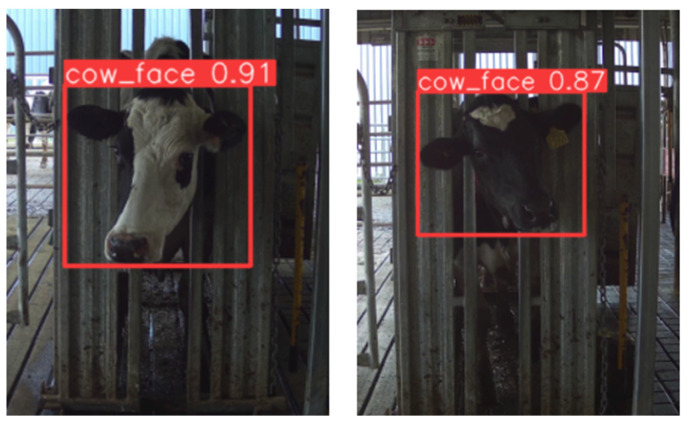
Example of face detection showing the confidence score.

The face detector searches for the cow’s frontal face in the input image. This model was developed based on YOLOv5 [20]. It localizes the frontal face position of the cow by returning the coordinates of four points, denoting a bounding box covering the face, along with a score within a [0, 1] range, implying the confidence level of the detected object. A value of 1 is an actual cow’s face, and 0 indicates it is less likely to be a cow’s face (Figure 3). A threshold is currently set as 0.7 to filter false detection, which means if the detected face has a score above 0.7, it will be post-processed for the next step, otherwise discarded, and moved on to the next frame. Although the video frame’s resolution is 4K, input images were resized to (640 × 360) in width and height to minimize the computation cost and power consumption when moving the program onto edge devices. In addition, the architecture of YOLOv5 used in this study was YOLOv5s6, where s6 denotes small model architecture, fifth version. The output class was reduced to one class, implying a cow’s face. During the training phase, the data augmentation techniques were randomly flipped along the *x*-axis (horizontal flip), mosaic [21], and random scale, which scales the image content at a random ratio while keeping the image size constant. All other hyperparameters were set to the default values given in YOLO’s repository (https://github.com/ultralytics/yolov5 (accessed on 10 September 2022)).

### 2.5. Landmark Predictor

**Figure 4:**
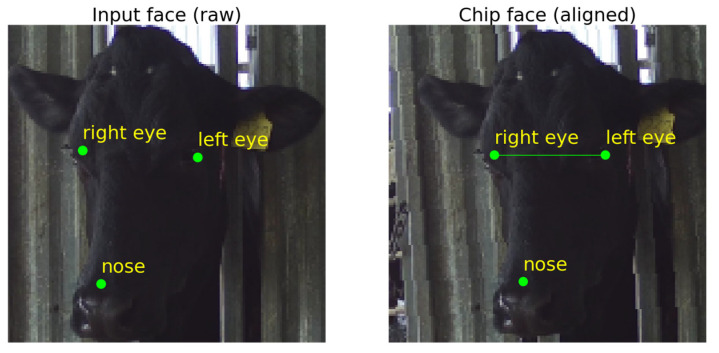
Example of facial landmarks prediction and aligned face.

Landmark predictor takes in the squared cropped face with a size of (224 × 224 pixels) and identifies specific facial key points: left eye, right eye, and the tip of the muzzle. These landmarks are anchors to align the face into the chip face for the Face encoder model (Figure 4). The Landmark predictor model was trained using Resnet18 [22] with means squared error loss (MSELoss) learning metric [23] and the Adam optimizer [24]. Parts of the implementation have been adapted from the “Face Landmark Detection with PyTorch” publicly available article [25].

### 2.6. Feature Encoder

**Figure 5:**
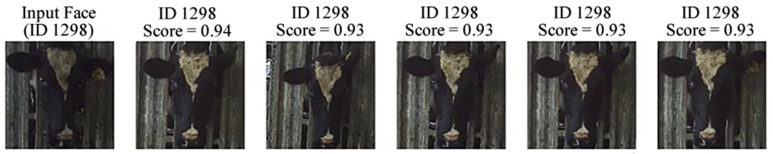
Example of input target face (first on the left) in a search returned top five most similar faces in the database.

Feature encoder plays a critical role in the pipeline that encodes the input chip face (a cropped and aligned face) from a size of 224 × 224 pixels into 512-embedding features, which are used to compare and search for similar faces in the database. The architecture includes two parts regarding the training phase: a backbone part used as a feature encoder and a head part used as a classifier during the training phase. Since the head part learns to classify a fixed set of IDs, it must be retrained every time a new ID is added to the system. So, the classifier layer of the head part (using softmax in the last layer as a classifier which is not preferred for face recognition problems [26]) will increase when more identities are available to be recognized. Consequently, the complexity will grow, and further retraining a deep learning model is computationally time-consuming. Therefore, the head part is used only during the training phase to help finetune the parameters of the backbone (feature encoder). The identification part handled by the face lookup step is more flexible and discriminative to novel faces because it bases on the similarity measurement (cosine similarity) to pick up the best-matched ID during the testing and inferencing (Figure 5). However, using the head part to perform the recognition task on the validation set can also be considered a valuable alternative baseline for comparison.

The backbone part, the feature encoder model, was developed using Resnet101 [22] to generate 512 embedding features. The head part used the ArcFace margin product network [26] that takes 512 embedding features from the backbone and outputs 89 ID classes. Parts of the ArcFace margin product network implementation have been adopted from the ArcFace-PyTorch repository (https://github.com/ronghuaiyang/arcface-pytorch (accessed on 10 September 2022)). This architecture was trained with the focal loss [27] learning metrics and cosine annealing [28] learning rate schedule. 

### 2.7. Database

A database was populated by 2225 chip faces representing 89 individual enrolled cow mates. The number of chip faces per identity (nim) was selected as the first 25 out of 40 chip faces per identity derived from the training dataset of the Face encoder. The corresponding embedding features of these chip faces were also recorded and made available to compute pairwise cosine similarity (1) with new chip faces. 

### 2.8. Evaluation Metrics

The pairwise similarity score between embedding features of the input chip face and other embedding features of enrolled cow mates in the database are measured by cosine similarity metric (Equation (1)), that bounds within the range [−1, 1] where “1” indicates a perfect match and “−1” means an exact opposite. In other words, the similar level increases when the similarity score runs from “−1” to “1”.

(1) $$cos\left(\theta \right)=\frac{A\cdot B}{\left\|A\|\|B\right\|}=\frac{\sum_{i=1}^{n}A_{i}B_{i}}{\sqrt{\sum_{i=1}^{n}A_{i}^{2}}\sqrt{\sum_{i=1}^{n}B_{i}^{2}}}$$

Regarding the Face detector model, mean average precision (mAP) is currently used as a standard metric for object detection models, also adopted by the series of YOLO models [29] and as the evaluation metric of the COCO dataset [18]. The *mAP* measures the mean of average precision for all categories. However, in this study, there was only one class, which was the cow’s face; therefore, *mAP@T* represents the precision of the model in detecting a cow’s face with an overlapping rate between the predicted bounding box and the ground truth bounding box that is greater than a threshold *T*. For example, *mAP@0.5* denotes the precision value at threshold *T* = 0.5. *mAP@0.5:0.95* is also popular that represents the mean precision over a set of precision values associated with the overlapping threshold range within 0.5–0.95 with a step of 0.05 [18]. Regarding the Landmark predictor model, mean squared error (MSE) [23] was used to measure the difference between the coordinates of predicted points concerning the original points.

Concerning the metrics for the Face encoder and the whole pipeline performance of the identification system in general, the following terminologies are used, derived from the Face recognition vendor test for the identification task of the National Institute of Standards and Technology US Department of Commerce [16]. According to the performance metrics under this standard, the evaluation of an identification system needs to be able to quantify two error conditions:

- False-positive: an error when a search is done for a non-mate cow, but the returned ID belonged to a mate cow.
- Miss: an error when a search is done for a mate cow, but the returned ID for that cow is out of the top R, or its score is below threshold *T*.

The false-positive cases accumulate to the false-positive identification rate (FPIR; Equation (2)), known as the “false alarm rate”, which shows the proportion of non-mate cow lookups that return an erroneous outcome given a specific threshold *T*

(2) $$FPIR\left(N,T\right)=\frac{number\;of\;false\;positives}{number\;of\;nonmate\;searches\;attempted}$$

The miss cases accumulate to the false-negative identification rate (FNIR; Equation (3)), known as the “miss rate”, which shows the proportion of unsuccessful mate cow searches given a specific threshold *T* and top R.

(3) $$FNIR\left(N,R,T\right)=\frac{number\;of\;misses}{number\;of\;mate\;searches\;attempted}$$

An opposite measurement for the “miss rate” is the “hit rate,” known as the true-positive identification rate (TPIR; Equation (4)).

(4) TPIR(N,R,T)=1−FNIR(N,R,T)

The general accuracy related to a mate search is called cumulative match characteristic (CMC; Equation (5)), which relaxes the similarity score threshold and only reports the proportion of successful mate searches.

(5) CMC(N,R)=1−FNIR(N,R,0)

Since the system is meant to operate autonomously without human supervision and in a controlled environment with a constant number of identities N for every search, the “rank one-hit rate” (TPIR with R = 1) was the accuracy metric used in this study to validate the performance of the whole pipeline in association with a specific threshold *T*.

A plot called detection error trade-off (DET), or identification error trade-off characteristic, is typically used to show two error types (FPIR and FNIR) on the same graph with a logarithmic scale. This plot helps compare the accuracy of the biometric system under different settings. Moreover, particularly in this study, it is mainly used to compare the system’s performance under different settings, such as the effect of enrolling in the database multiple images per identity and different buffer size settings. Another plot involving FPIR and FNIR is used to show the error rate across a range of threshold values, which helps select a threshold, especially in an environment where both enrolled cow mates and non-mates coexist. In this plot, an equal error rate (EER) point suggests a reference threshold *T* for the identification system, and the EER point is defined by the intersection of the FPIR and FNIR lines.

The matching speed measures the overall inference time of the pipeline over a static input image. This matching speed is essential in assessing whether the pipeline can run in real-time and measuring the speed improvement when different deep learning models are used.

## 3. Results and Discussion

### 3.1. Model Performance

The Face detector model achieved *mAP@0.5:0.95* = 0.95, which means the model’s average precision in predicting the location of a cow’s head correctly is 95% over a set of precision values corresponding to the overlapping ratio range between 0.5 and 0.95. 

The Landmark predictor achieved an MSE of 9.2, equivalent to a prediction point with an error swing within a circle with a radius = 3 (square root of 9.2) in pixel, centered at the ground truth point. This MSE value equals a 1.35% error rate (3/224-pixel width).

**Figure 6:**
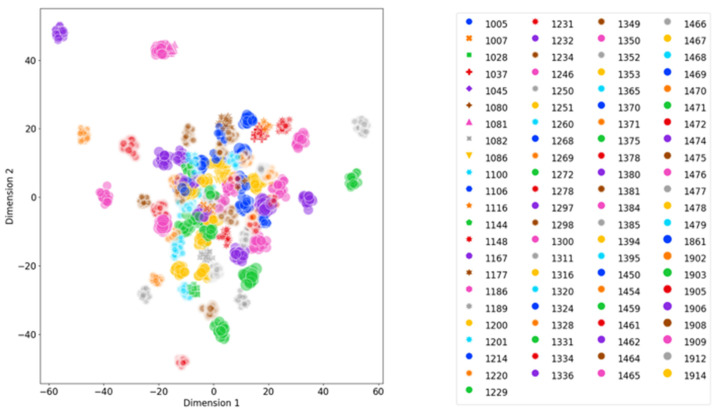
Cow ID distributed in 2-dimensional feature space with linear discriminant analysis processing.

Regarding the performance assessment of the Face encoder, two of the following architectures were compared. The first method structure used in the training phase, backbone and head part, recorded CMC (N = 89, R = 1) = 84.55% accuracy on the validation set. Besides, using the Face encoder’s backbone and the face lookup step resulted in CMC (N = 89, R = 1) = 93.14% accuracy on the validation dataset. Therefore, it is recommended to use the backbone as a feature extractor. Furthermore, using the generated features to perform cosine similarity is preferred and used as the overall pipeline performance. Figure 6 shows a distribution map of chip face embedding features in 2D space of all enrolled cow mates in the database using linear discriminant analysis (LDA) [30] techniques with pre-processing steps, including feature scaling and normalization. It can be seen that chip faces of the same identity are grouped into separate clusters. There is still room to include more identities and chip faces in the database without overpopulating the 512-dimensional space.

Up to this point, all three deep learning models were finetuned well to the cow’s facial features and achieved good results. The pipeline on the validation set also showed a good result. The next step was to assess the whole identification system on two test sets: the open set and the closed set in the form of still images and video sequences.

### 3.2. Cows Identification on Still Images

The cow identification system was tested on two test sets to validate two error types in this experiment. The first set, referred to as the closed set, contained only the ID of enrolled cow mates in the database, and it was used to quantify the FNIR or miss rate. The second set, referred to as open-set, contained only the ID of cows not included in the database to quantify the FPIR or false alarm rate.

**Figure 7:**
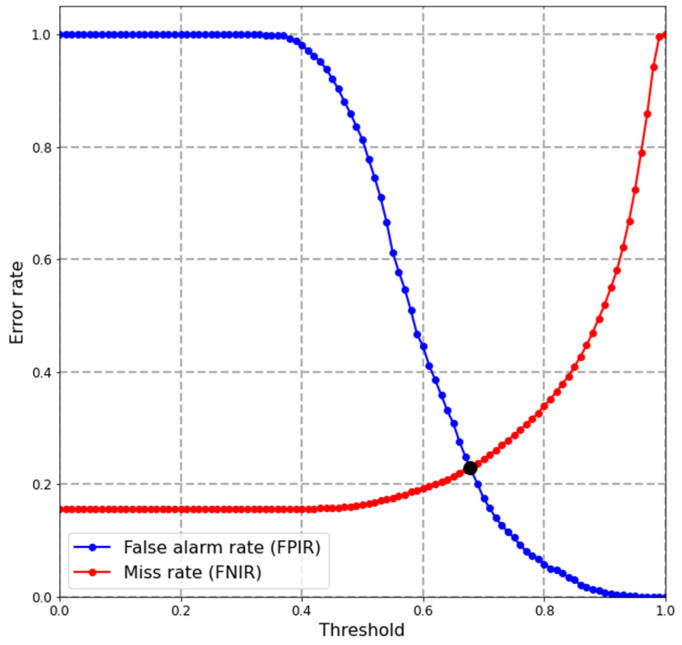
Detection error trade-off (number of identity N = 89, top R = 1, number of images per identity *nim* = 25) characteristics of identification system on test set.

The following results were conducted with 25 chip images per identity (*nim* = 25) and considering just the top one ranking (R = 1). With a relaxed threshold *T* = 0, the CMC value derived from the closed-set was 84%, equivalent to an accuracy of a hit rate of 84% with only two cows that were missed entirely from all of its test samples. The latter can be inferred as 87 out of 89 individuals were correctly identified by the system. By introducing a range of threshold values and testing on both open-set and closed-set, FPIR and FNIR rates were obtained to visualize the system’s overall detection error trade-off characteristic (Figure 7).

**Figure 8:**
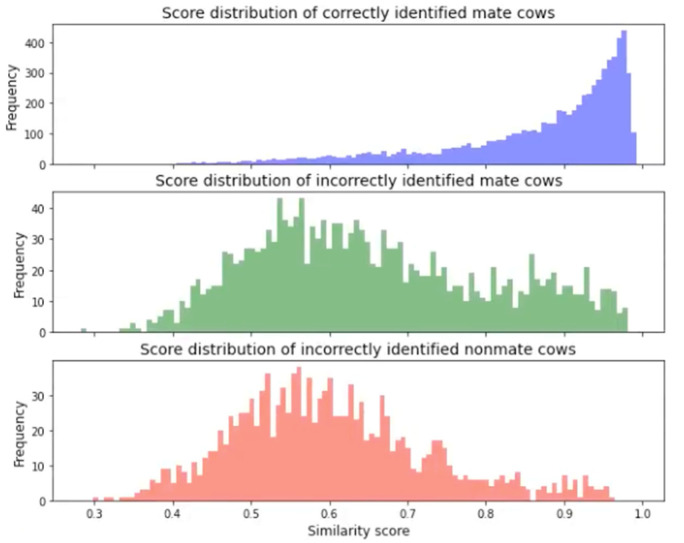
Score distribution in comparison between mate and non-mate cows from closed-set and open-set, respectively.

**Figure 9:**
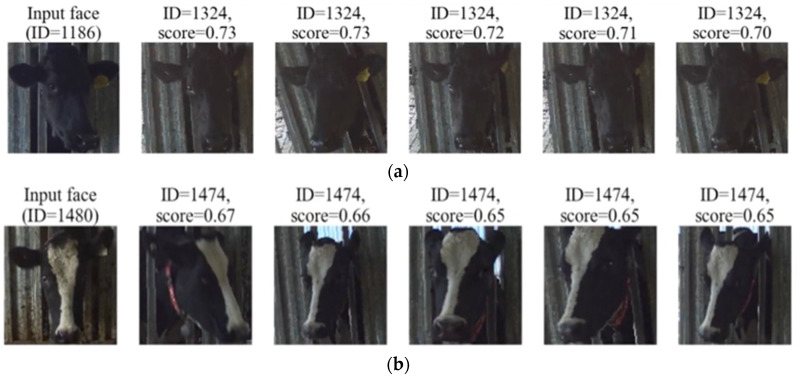
Example of (**a**) a miss case (ID = 1186 enrolled in database) and (**b**) a false alarm case (ID = 1480 not in database) in which input face looks very similar to an enrolled identity.

Looking at the score distribution histogram of all the top one ranking prediction IDs (Figure 8), a mate search score distribution reaches a peak near value “1”, which is consistent with the high similarity-score values. However, many correctly identified pairs had low confidence scores (long-tail). Similarly, many pairs had relatively high confidence in the identification result for the unmatching mate searches. The latter means that some individuals closely resemble each other in color and facial patterns (Figure 9). A similar phenomenon was also recorded in the score distribution of non-mate searches when the input face looked similar to some enrolled identities. Figure 9 shows an example of a missing case and a false alarm case where the input face looked similar to an enrolled identity.

Suppose the identification system needs to identify both enrolled mate and non-mate cows. To select an appropriate threshold, it is more practical to look at the score distribution (Figure 8) or the error rate against the threshold graph (Figure 7). For example, a threshold with a lower value can be used if the case study requires a low miss rate and does not need to detect unenrolled faces. In contrast, if the case study requires a harmonious balance between the false alarm rate and miss rate, it is practical to obtain the equal error rate (EER) point where FPIR = FNIR. From Figure 7, the EER point was 0.23, the threshold *T* was set to 0.68, and the corresponding hit rate TPIR (89, 1, 0.68) achieved 77% accuracy. Otherwise, if only enrolled mate cows are subjects of the identification system, and no non-mate face needs to be detected, it is possible to relax the threshold to *T* = 0. The cumulative match characteristic value should be the metric used.

**Figure 10:**
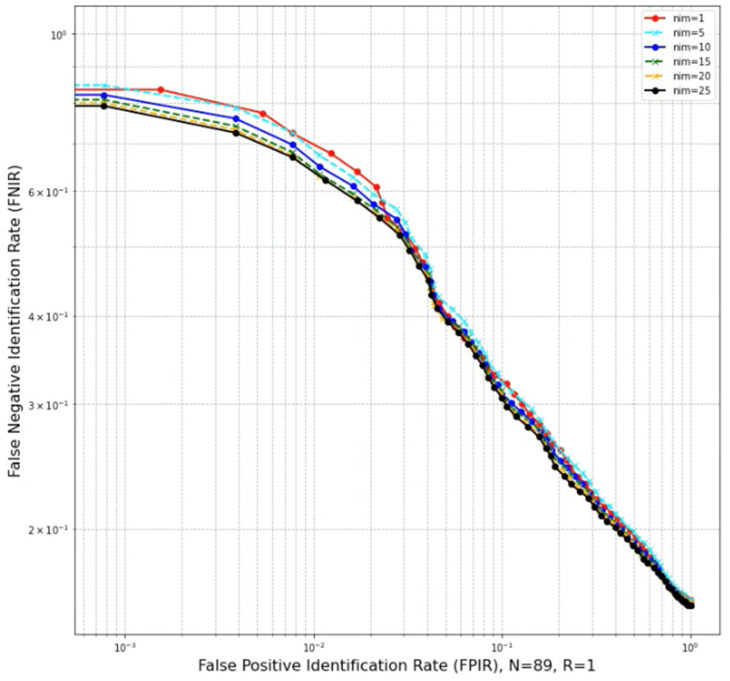
Effect of enrolling multiple images for each identity.

Regarding processing video sequences, a detected face may come from various angles. Having more representative chip faces with diverse views can help improve the overall performance of the identification system [31]. Therefore, an experiment was conducted to evaluate the effect of different pipeline settings in the events where the number of enrolling images per identity (*nim*) ranges from 1 to 25 with a step of 5, the number of enrolled mate N = 89, and top R = 1. The experimental result supported the above statement, as increasing the *nim* decreases the miss rate (Figure 10).

This study also tested different numbers of match IDs returned by the pipeline, known as different top R values. Accordingly, a human or an automated process must select the final ID if the candidate list is returned. In this study, an automated process was tested to select the top ID using the top R *=* 5 and R = 10 as a voting mechanism, returning as the final prediction of the ID with the most occurrences in the returned candidate list. The cumulative match characteristic of each combination of (R, *nim*) is shown in Table 1 to examine the effect of different top R settings associated with various images per identity. The CMC values at a fixed top R increased when the database expanded from having only one representative chip face to having more. However, when the *nim* value is larger, their chance of having a mismatched pair is higher, which explains why the CMC value drops slightly when *nim* increases. Note that with a fixed *nim* value, increasing the length of the candidate list incurs a slightly improved CMC value.

Finally, the matching speed measurement is also essential for the system setup and configuration, necessary for the deployment phase. The pipeline takes ~100 ms to process a still input image (Table 2). The recorded inference speed can be considered a baseline for future development and improvement. It is possible to run the system in real-time and process up to 10 fps with the current recorded speed.

### 3.3. Cows Identification on Video Sequences

Not all frames were processed to test the identification system on a video sequence, which is time-consuming. In addition, it is possible to treat each frame as a still image; however, it is easy to encounter a missing case between a sequence of hit cases, so in such case, there is a flicker in the result indication shown on the monitor. Therefore, a buffer was introduced to help stabilize the returned ID. The buffer integrates past information (detected pipID from previous frames) with the latest one (detected pipID from the latest frame) using a voting mechanism similar to the one used on still images with R = 5 and R = 10. The bigger the buffer size, the more time is needed to fill it with frames.

**Figure 11:**
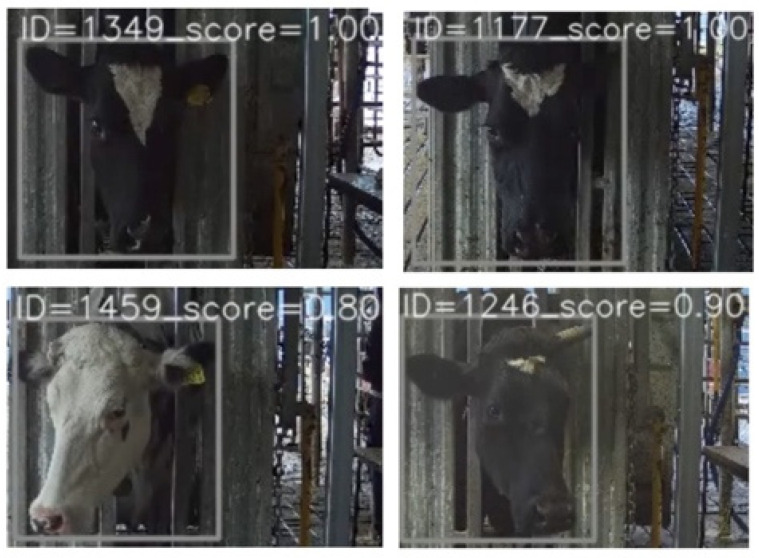
Example of visualized identified cows with bufID and bufScore processed on video sequence with buffer size of 10.

**Figure 12:**
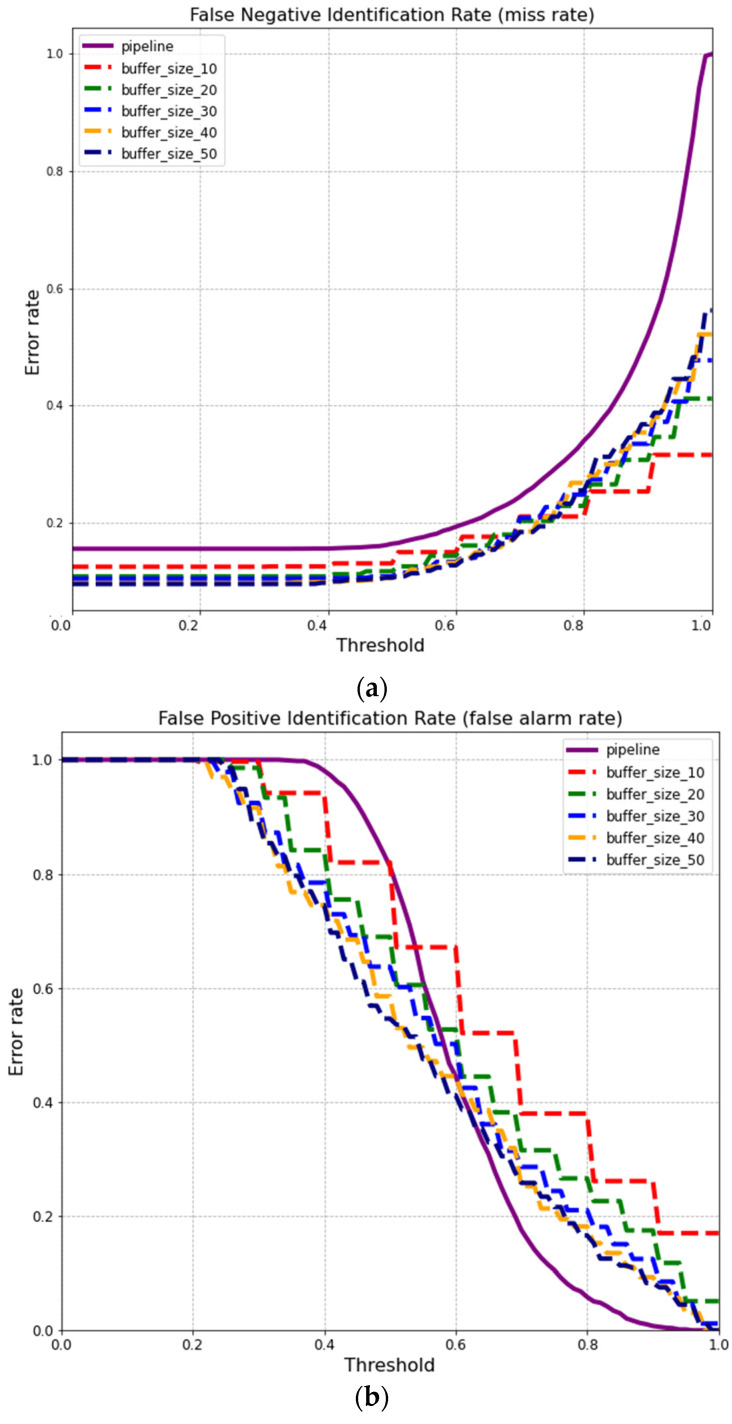
Comparison of detection error trade-off characteristic lines between various buffer sizes and pipeline settings for the (**a**) false negative rate and (**b**) false positive rate.

Using both the open-set and closed-set, the detected ID returned by the pipeline (pipID) for each frame is added to the buffer until full. Once the buffer is filled up, it will indicate on the monitor the most occurrent ID in the buffer, and the associated score is the proportion frames where that ID appeared in the buffer (Figure 11), denoted as bufID and bufScore, respectively. For example, in Figure 12, the buffer size is 10, and a score of 0.9 implies ID = 1246 has been predicted over 90% of the frames in the buffer. When the next pipID is added to a full buffer, the oldest pipID in the buffer will be discarded to make room for the upcoming pipID. A threshold designated for this buffer can also be introduced, called bufThreshold, to filter out less confident ID via the bufScore. With the use of bufThreshold, the evaluation metrics such as FPIR, FNIR, and CMC values can be obtained similarly to the values obtained on still images.

In order to show the advantage of a buffer in processing video sequences over still images, FPIR and FNIR values were measured using the same settings as the single image pipeline (R = 1, *nim* = 25, *T* = 0) along with different buffer sizes (Figure 12). Regarding the false-negative identification graph in Figure 12, the buffer implementation is helpful in reducing the miss rate regardless of buffer size and the threshold value. Therefore, in a closed-set scenario, where no new face is recognized by the system, implementing a buffer helps to boost performance. Implementing a buffer for video processing does not always guarantee a lower error rate regarding an open-set scenario.

As shown in Table 3, a larger buffer size also helps improve the CMC hit rate recorded concerning the single image pipeline, jumping from 84% to 88% and 91%, with buffer sizes ranging from 10 to 50. At the same time, the number of unique mate IDs returned correctly for all mate search attempts (true-positive) results in a slight decline in the #Mate ID true-positive from 87 IDs down to 86 and 84 for buffer sizes from 10 up to 50. This phenomenon occurs due to the stabilization effect of the buffer that filtered out true-positive cases of mate IDs that are less similar to their representative chip faces in the database and more similar to other enrolled cow mates. Regarding non-mate search, this stabilization effect of the buffer shows a positive result that helps minimize the recorded number of unique false-positive mate IDs over all the non-mate search attempts, from 52 down to 42 and 19 as for buffer size from 10 up to 50. However, regardless of this minor drop, the overall system gained more confidence in its prediction as the CMC results increased. Even when the top R-value increased from one to five and 10, a similar positive effect was observed due to increasing the buffer size. However, increasing the top R-value while keeping the buffer size constant may not improve performance.

An optimal choice of buffer size depends on how it will behave in the field test at the deployment stage. It is a trade-off between performance and matching speed.

### 3.4. Deployment

**Figure 13:**
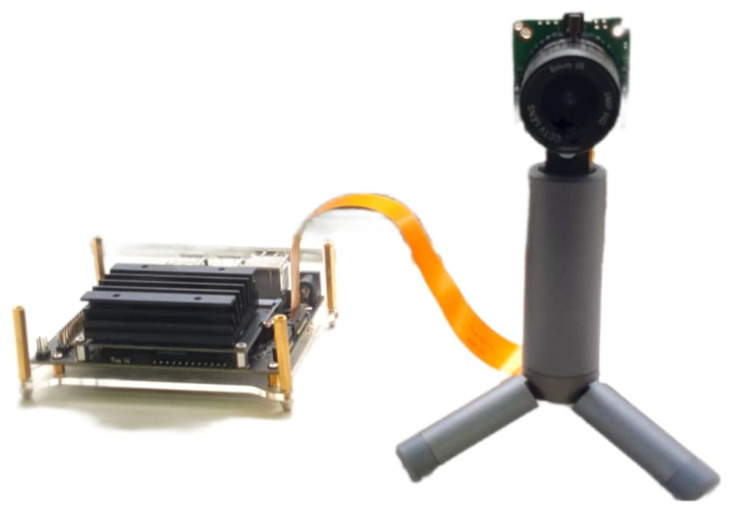
Hardware setup for system deployment using NVIDIA Jetson nano and a 12 MP camera.

The deployment phase integrated the deep learning models into an NVIDIA Jetson Nano (NVIDIA, Santa Clara, CA, USA) using a 12 MP camera (Figure 13). This board is equipped with a 128-core GPU that delivers up to 472 GFLOPs of computing power, which is enough to yield real-time identification of cows. This camera can be installed at the farm to monitor and identify cows at any point that farmers or producers consider convenient. It aims to place it in a location that mimics the angle the cow’s models were trained. A proposal setup can be by integrating the camera at the gate of the squeeze chute to simplify the number of cows to be identified to just one at a time. One limitation of the proposal may be the short length of the camera’s ribbon cable. However, the board does support other camera interfaces such as USB or TCP/IP video stream via network. Not to mention that the board can host a website and exposes its IP address to other devices in the same local network to interact with the face ID recognition program. If applicable, any smartphone with a built-in camera can snap, send, and retrieve the cow’s ID on its web browser. Further field test is required to validate an optimal setup (e.g., camera positioning, power supply, and connectivity) as well as to evaluate external environment factors (e.g., lightning, dust, ambient temperature). The deployed device can incorporate other algorithms to predict physiological responses [32].

Since the number of NVIDIA Jetson nano GPU cores is much lower than GeForce GTX 2080 graphic card used in testing (which has 23 times more computing cores), the inference time of the Jetson nano is much slower, about 6.5 times slower (Table 4). So as not to interrupt the video streaming processing on the main thread, the pipeline was redesigned to be triggered on a new thread whenever a new frame is sampled. With that said, the main processing thread is handling video streaming. The newly triggered thread will process the recognition task without interfering with the video streaming of the main thread. To prevent the system from throttling when another recognition thread is triggered while the previous recognition thread has not been completed, the number of new threads running alongside the main thread is limited to only one at a time and not a queue. This implementation allows the video streaming to run at real-time speed while there is a minor lagging for the recognition result to be indicated on the monitor. The lagging is approximately 600 ms for processing the recognition task and returning the result (ID and score), as measured in Table 4.

In an ideal scenario, it is expected that a non-mate cow search would return a low similarity score against all the enrolled cow mates. However, as experiments showed, false alarm cases with a relatively high similarity score still exist. The farmer will usually know which cow is newly transferred to their farm. It is recommended to manually capture a few samples of a non-mate cow and add the encoding to the database. When adding new information to the database, retraining any deep learning models is unnecessary, although retraining the face encoder may improve performance.

Similarly, when users need to remove a cow from the database, they must delete the corresponding chip faces and remove embedded features from the database. Regarding a missing case, when the returned ID has a low confidence score, the user should manually verify the ID of that cow, either via the attached ear tag or other preferred identification methods. A systematic study will be conducted to determine the best protocol for data acquisition in future work.

### 3.5. Discussion

In 2020 the average herd size of an Australian dairy farm had grown to an average of 279 head [33]. Although the number of unique identities experimented within this study is small compared to standard industrial herd size, the ArcFace algorithm showed high accuracy on various datasets containing thousands to millions of identities [26]. With the spacious 2D cow feature space (Figure 6), there is still capacity for this system to take up more identities and scale up to an industrial size.

Working with computer vision systems often encounters external impacts from the environment to the overall performance, such as occlusion, illumination, or background problems. Since no obstacles were recorded between the camera and the cow across all videos in the dataset, the occlusion problem was not addressed in this paper. Regarding the illumination problem, although all videos were recorded in good lighting conditions, image augmentation methods were applied to simulate varied input image colors (e.g., randomly adjust brightness, contrast, saturation, and gaussian blur), minimizing the effect of illumination changes. However, further testing the impact of either occlusion or illumination of the environment during field tests can be addressed in future work. One concern about the current implementation of the pipeline is the chip face’s background impact on the performance of the face lookup. Namely, the system is sensitive to minor background changes, even though many data augmentation steps were used while training each deep learning model. For example, in some “chip face” samples, a tool supports the cow’s head. Since the device is yellow, when part of the tool is captured in the chip face, it may affect the prediction accuracy when the same cow is recognized without the device occluding its face. Likewise, chip faces of another identity with the yellow neck support tool may also likely be mispredicted with those in the database. Therefore, it is intended to blur or filter the background to decrease its impact on the pipeline in the future.

Assuming a well-controlled environment on a farm scale is equivalent to a closed-set, where all cows have been enrolled into the database. Consequently, the identification system will have no chance to see a non-mate cow, so the only metric needed is the CMC value. Alternatively, a hit rate value that maps to a specific threshold metric should be used.

An advantage of the model developed is that it can be integrated with previous works from the Digital Agriculture, Food and Wine research group from The University of Melbourne related to the welfare assessment of farm animals. Specifically, different models have been developed to extract animal physiological information from RGB and infrared thermal videos, such as heart rate, eye temperature, respiration rate, and sudden movements for pigs [34], sheep [35], cattle [36], and dairy cows [32]. Hence, integrating the analysis pipeline (Figure 2) up to face cropping with the ML models developed for animal physiology will allow extracting the ID per animal and parameters to assess welfare. These integrated models may not require a FLIR DUO PRO, just normal RGB cameras, since eye temperature has been extracted using ML models from heart rate and respiration rate plus environmental temperature and relative humidity for dairy cows [32]. The latter explains using the FLIR visible and infrared thermal integrated camera for this study. However, the deployment option proposed will be the same as in Figure 13.

By integrating ID and welfare ML models, the industry can have further advantages related to predictions of the quality of produces related to individual animals and their physiological responses to the environment, such as quality and composition of milk [32] and meat quality for cattle [36] and early detection of respiratory problems for pigs [34].

## 4. Conclusions

This study presented a deep-learning facial recognition pipeline on dairy cows with high accuracy and deployment options. Furthermore, the pipeline proposed is easy to upgrade as its fundamental steps are segmented into smaller modules. The steps to add or remove an identity to/from the database would not need to retrain other models. The deployment test on the Jetson nano board showed that the system could be portable and used with mobile devices such as smartphones and tablet PCs. This identification system can then be integrated with other technology, such as biometric tracking, for animal welfare monitoring purposes. It also showed that existing machine learning tools are accessible, advanced, and easy to use for various purposes by end-users in both industry and academia. Importantly, this pipeline and method are non-invasive to the animal, quick, and reliable. It can complement the conventional checking of every animal based on ear tags or paper checklists if required. The system can be used for other farm animals, requiring only retraining, which can be a significant leap for livestock monitoring, not only for traceability but also to track the welfare of animals on the farm during transport and avoid fraudulent practices, and predict the quality of produces, such as milk and meat and the effects of environmental stresses, such as heat stress on produces.

## Figures and Tables

**Table 1:** Cumulative match characteristic (%) of selecting different returned Top R.

*nim*	Top R		
	1	5	10
1	84.15	-	-
5	84.21	84.27	-
10	84.35	84.25	84.35
15	84.35	84.27	84.30
20	84.34	84.31	84.35
25	84.42	84.33	84.31

Abbreviations: *nim*: number of images per identity; Top R: top closest candidate to examine.

**Table 2:** Timing measurement of the pipeline inferencing with NVIDIA GeForce GTX2080 graphic card.

Step	Inference Speed
Face detection	23.4 ms
Face cropping	7.3 ms
Face encoding	24.9 ms
Face lookup	42.9 ms
Total	~98.5 ms

**Table 3:** Comparing effect of buffer size on performance on video sequences.

Number of Images per Identity *nim* = 25	Top R	Pipeline	Buffer Size				
			10	20	30	40	50
CMC (%)	1	84	88	89	90	90	90
	5	84	88	89	90	90	91
	10	84	88	89	90	90	91
#Mate ID true positive	1	87	86	86	85	86	85
	5	87	86	86	85	85	85
	10	87	87	85	84	84	84
#Nonmate ID false positive	1	52	42	34	32	25	19
	5	52	42	34	31	25	23
	10	52	42	35	35	25	21

Abbreviations: CMC: cumulative match characteristic; *nim*: number of images per identity; Top R: top closest candidate to examine.

**Table 4:** Matching speed of the pipeline running on Jetson Nano.

Step	Inference Speed
Face detector	177.1 ms
Face cropper	47.9 ms
Face encoder	177.7 ms
Face lookup	236.8 ms
Total	~639.5 ms

## Data Availability

Data and intellectual property belong to The University of Melbourne; any sharing needs to be evaluated and approved by the University.

## References

[1] Kumar S., Singh S.K. (2020). Cattle recognition: A new frontier in visual animal biometrics research. Proc. Natl. Acad. Sci. India Sect. A Phys. Sci..

[2] Kumar S., Tiwari S., Singh S.K. (2016). Face Recognition of Cattle: Can it be Done?. Proc. Natl. Acad. Sci. India Sect. A Phys. Sci..

[3] Zin T.T., Phyo C.N., Tin P., Hama H., Kobayashi I. Image technology based cow identification system using deep learning. Proceedings of the International MultiConference of Engineers and Computer Scientists.

[4] Kumar S., Singh S.K., Singh R., Singh A.K. (2017). Muzzle point pattern based techniques for individual cattle identification. IET Image Process..

[5] Awad A.I. (2016). From classical methods to animal biometrics: A review on cattle identification and tracking. Comput. Electron. Agric..

[6] Stärk K.D.C., Morris R.S., Pfeiffer D.U. (1998). Comparison of electronic and visual identification systems in pigs. Livest. Prod. Sci..

[7] Bergqvist A.-S., Forsberg F., Eliasson C., Wallenbeck A. (2015). Individual identification of pigs during rearing and at slaughter using microchips. Livest. Sci..

[8] Nason J. (2020). Tag Retention: NLIS Tag Losses Still Frustrating Producers. https://www.beefcentral.com/news/tag-retention-nlis-tag-losses-still-frustrating-producers/#:~:text=CATTLE%20producers%20are%20reporting%20high,of%20sale%20for%20traceability%20purposes..

[9] Clapham M., Miller E., Nguyen M., Darimont C.T. (2020). Automated facial recognition for wildlife that lack unique markings: A deep learning approach for brown bears. Ecol. Evol..

[10] Mazzeo P.L., Xue H., Qin J., Quan C., Ren W., Gao T., Zhao J. (2021). Open Set Sheep Face Recognition Based on Euclidean Space Metric. Math. Probl. Eng..

[11] Matkowski W.M., Kong A.W.K., Su H., Chen P., Hou R., Zhang Z. Giant Panda Face Recognition Using Small Dataset. Proceedings of the 2019 IEEE International Conference on Image Processing (ICIP).

[12] Cai C., Li J. Cattle face recognition using local binary pattern descriptor. Proceedings of the 2013 Asia-Pacific Signal and Information Processing Assiciation Annual Summit and Conference.

[13] Lu Y., He X., Wen Y., Wang P.S.P. (2014). A new cow identification system based on iris analysis and recognition. Int. J. Biom..

[14] Phillips P., Beveridge J., Givens G., Draper B., Lui Y., Bolme D. (2013). Introduction to Face Recognition and Evaluation of Algorithm Performance. Comput. Stat. Data Anal..

[15] Intel Corporation (2018). Toolkit, O. cvat.

[16] Grother P., Ngan M., Hanaoka K. (2019). Face Recognition Vendor Test (FRVT) Part 2: Identification.

[17] Deng J., Dong W., Socher R., Li L.-J., Li K., Fei-Fei L. Imagenet: A large-scale hierarchical image database. Proceedings of the 2009 IEEE Conference on Computer Vision and Pattern Recognition.

[18] Lin T.-Y., Maire M., Belongie S., Bourdev L., Girshick R., Hays J., Perona P., Ramanan D., Zitnick C.L., Dollár P. (2014). Microsoft COCO: Common Objects in Context. European Conference on Computer Vision.

[19] Sandler M., Howard A., Zhu M., Zhmoginov A., Chen L.-C. MobileNetV2: Inverted Residuals and Linear Bottlenecks. Proceedings of the IEEE Conference on Computer Vision and Pattern Recognition (CVPR).

[20] Jocher G. Ultralytics YOLOv5, GitHub: 2021. https://github.com/ultralytics/yolov5.

[21] Bochkovskiy A., Wang C.-Y., Liao H.-Y.M. (2020). YOLOv4: Optimal Speed and Accuracy of Object Detection. arXiv.

[22] He K., Zhang X., Ren S., Sun J. (2015). Deep Residual Learning for Image Recognition. arXiv.

[23] Sammut C., Webb G.I. (2010). Mean Squared Error. Encyclopedia of Machine Learning.

[24] Kingma D.P., Ba J. (2014). Adam: A Method for Stochastic Optimization. axRiv.

[25] Kalim A.R. (2020). Face Landmarks Detection with PyTorch.

[26] Deng J., Guo J., Xue N., Zafeiriou S. ArcFace: Additive Angular Margin Loss for Deep Face Recognition. Proceedings of the IEEE/CVF Conference on Computer Vision and Pattern Recognition.

[27] Lin T.-Y., Goyal P., Girshick R., He K., Dollár P. (2018). Focal Loss for Dense Object Detection. arXiv.

[28] Loshchilov I., Hutter F. (2017). SGDR: Stochastic Gradient Descent with Warm Restarts. arXiv.

[29] Redmon J., Divvala S., Girshick R., Farhadi A. (2015). You Only Look Once: Unified, Real-Time Object Detection. arXiv.

[30] Fisher R.A., Li S.Z., Jain A. (2009). LDA (Linear Discriminant Analysis). Encyclopedia of Biometrics.

[31] Grother P., Quinn G., Ngan M. (2017). NIST Interagency Report 8173: Face in Video Evaluation (FIVE) Face Recognition of Non-Cooperative Subjects.

[32] Fuentes S., Viejo C.G., Tongson E., Lipovetzky N., Dunshea F.R. (2021). Biometric Physiological Responses from Dairy Cows Measured by Visible Remote Sensing Are Good Predictors of Milk Productivity and Quality through Artificial Intelligence. Sensors.

[33] Dairy Australia Cow & Farms Data. https://www.dairyaustralia.com.au/industry-statistics/cow-and-farms-data#.Y1skeHZBw2w.

[34] Jorquera-Chavez M., Fuentes S., Dunshea F.R., Warner R.D., Poblete T., Morrison R.S., Jongman E.C. (2020). Remotely Sensed Imagery for Early Detection of Respiratory Disease in Pigs: A Pilot Study. Animals.

[35] Fuentes S., Viejo C.G., Chauhan S.S., Joy A., Tongson E., Dunshea F.R. (2020). Non-Invasive Sheep Biometrics Obtained by Computer Vision Algorithms and Machine Learning Modeling Using Integrated Visible/Infrared Thermal Cameras. Sensors.

[36] Jorquera-Chavez M., Fuentes S., Dunshea F.R., Warner R.D., Poblete T., Jongman E.C. (2019). Modelling and validation of computer vision techniques to assess heart rate, eye temperature, ear-base temperature and respiration rate in cattle. Animals.

